# Synchronization and spontaneous dynamics in the Locus Coeruleus network

**DOI:** 10.1186/1471-2202-12-S1-P219

**Published:** 2011-07-18

**Authors:** Georgi S Medvedev

**Affiliations:** 1 Department of Mathematics, Drexel University, Philadelphia, PA 19104, USA

## 

The mathematical theory of pattern formation in electrically coupled networks of excitable neurons forced by small noise is presented in this work. Using the Freidlin-Wentzell large deviation theory for randomly perturbed dynamical systems and the elements of the algebraic graph theory, we identify and analyze main regimes in the network dynamics in terms of the key control parameters: excitability, coupling strength, and network topology. The analysis reveals the geometry of spontaneous dynamics in electrically coupled network. Specifically, we show that the location of the minima of a certain continuous function on the surface of the unit n-cube encodes the most likely activity patterns generated by the network. By studying how the minima of this function evolve under the variation of the coupling strength, we identify the principal transformations in the network dynamics. The minimization problem is also used for the quantitative description of the main dynamical regimes and transitions between them. In particular, for the weak and strong coupling regimes, we present asymptotic formulae for the activity rate as a function of the coupling strength and the degree of the network. The variational analysis is complemented by the stability analysis of the synchronous state in the strong coupling regime. The stability estimates reveal the contribution of the network connectivity and the properties of the cycle subspace associated with the graph of the network to its synchronization properties. This work is motivated by the experimental and modeling studies of the ensemble of neurons in the Locus Coeruleus, a nucleus in the brainstem involved in the regulation of cognitive performance and behavior [1].
